# Ice cleat distribution programmes and ice cleat use among older adults: repeated cross-sectional evidence from 63 municipal interventions in Sweden

**DOI:** 10.1136/ip-2022-044681

**Published:** 2022-08-03

**Authors:** Robin Holmberg, Johanna Gustavsson, Mikael Svensson, Carl Bonander

**Affiliations:** 1 Department of Political, Historical and Cultural Studies, Karlstad University, Karlstad, Sweden; 2 Centre for Societal Risk Research, Karlstad University, Karlstad, Sweden; 3 School of Public Health and Community Medicine, Institute of Medicine, University of Gothenburg Sahlgrenska Academy, Gothenburg, Sweden

**Keywords:** behavior change, uptake/adherence, fall, process/impact evaluation, cross sectional study, older people

## Abstract

**Introduction:**

Ice cleats may help prevent ice-related falls in places with icy roads, but there is limited evidence about the association between ice cleat distribution and ice cleat use. Our study examined the association between Swedish municipal distribution programmes and ice cleat use among older adults (65+ years).

**Methods:**

We combined data on municipal ice cleat distribution programmes (n=63) with repeated cross-sectional self-reports of ice cleat use in Sweden from 2007, 2010, 2014 and 2018. Respondents (n=63 234) were classified as exposed if they lived in a municipality with a programme, belonged to an eligible age group and responded after distribution (n=2507). Dose-response was assessed using distributed ice cleat pairs per capita (mean: 0.38). Linear probability models were used to estimate probability differences in ice cleat use between exposed and unexposed respondents, adjusting for age, sex, country of birth, education, survey wave and municipality. Ineligible age groups living in programme municipalities, who should be unaffected by ice cleat distribution, were used for bias assessment.

**Results:**

Exposure to ice cleat distribution programmes was associated with 7.5 percentage points (95% CI 4.2 to 10.9) higher self-reported ice cleat use after confounding adjustment. The association was larger in municipalities that distributed one pair of ice cleats per capita (17.3 percentage points (95% CI 11.2 to 23.4)). No association was found among the ineligible age groups (−2.3 (95% CI −5.5 to 1.0)).

**Conclusion:**

Distributing ice cleats to older adults may help increase their use of ice cleats in settings with icy road conditions.

**WHAT IS ALREADY KNOWN ON THIS TOPIC**

- Previous research reports that the distribution of a pair of free anti-slip devices (ice cleats) to older adults (65+ years) may decrease ice-related fall injuries. Such distribution programmes are also low cost and likely to require minimal behaviour change to be cost-effective. However, there is currently no direct evidence that distribution influences ice cleat use, which means that the mechanisms through which ice cleat distribution affects ice-related injuries can be questioned.

**WHAT THIS STUDY ADDS**

- We find an association between ice cleat distribution in Swedish municipalities and increased ice cleat use among older adults targeted by the programmes. The findings also show that greater reach (distributed ice cleats per capita) is associated with greater increases in ice cleat use.

**HOW THIS STUDY MIGHT AFFECT RESEARCH, PRACTICE OR POLICY**

- Our study suggests that targeted ice cleat distribution by local governments may increase ice cleat use among older adults in settings with icy road conditions. While confirmatory trials are required before drawing strong causal conclusions, our study provides evidence for decision making that may help improve pedestrian safety for older adults.

## Introduction

Falls are the most frequent cause of injury among older adults.^1^ Environmental factors can affect the risk of falling. One such risk factor is recurrent exposure to ice and snow, which in the northern parts of the world increases the risk of fall-related injuries during winter.^2–4^ Ice cleats—anti-slip devices applied to shoes to improve grip on icy or snow-covered surfaces—can reduce the risk of fall injuries during winter conditions.^5–7^ Increasing the use of ice cleats in older populations could thus help combat the seasonal rise in ice-related fall injuries.

Many Swedish municipalities have begun distributing ice cleats to older adults to alleviate the increased risks of pedestrian falls associated with icy road conditions.^8^ However, these programmes vary in design in ways that are likely to influence their reach^9^ and impact on behaviours. For instance, some municipalities mailed coupons to senior citizens that could be exchanged for a free pair of ice cleats at designated stores, some offered a free pair of devices when eating lunch at any municipal-owned restaurant, and in others, municipal officials handed out ice cleats during fall prevention events.^8^

It is well established that ice cleats can reduce ice-related injury risks at the individual level,^5–7 10^ but data on the effects of ice cleat distribution is relatively scarce. One study from Gothenburg, Sweden, found a reduction in emergency department visits due to ice-related fall injuries during the first year after ice cleats were distributed by the municipality.^11^ From an economic perspective, simulations based on input data from Swedish ice cleat programmes also indicate that a very small increase in ice cleat use (0.15% of the population if one ice cleat pair is purchased for each citizen) could be sufficient for the programmes to pass a cost–benefit test at the societal level, due to low programme costs.^12^ While ice cleat distribution programmes seem reasonable from an injury and economic perspective, whether these programmes influence usage rates have yet to be established empirically.^8^ It is also unclear whether greater reach (ie, more ice cleat distributed per citizen) leads to greater increases in ice cleat use. Therefore, this study aimed to investigate the association between municipal ice cleat distribution programmes and ice cleat use and assess the dose–response relationship between ice cleat use and the number of ice cleats distributed per capita.

## Materials and methods

In this repeated cross-sectional study of the association between ice cleat distribution and ice cleat use, we collected data on municipal ice cleat distribution programmes and combined them with data on self-reported ice cleat use in the Swedish population aged 18–79 years.

### Data collection

#### Programme data

As part of a previous process evaluation study of ice cleat distribution in Sweden,^8^ we conducted an electronic survey sent to all Swedish municipalities (n=290) in 2019 to collect data about ice cleat programmes. In total, 228 municipalities participated and were asked if they had ever distributed ice cleats. If they said yes, we asked for further information on programme characteristics. Detailed characteristics of the programmes, including communication strategies, distribution points, programme duration, costs, types of ice cleats distributed and programme theory, have been described previously.^8^ For this study, we used municipality-level data on the year of implementation and age of eligibility to classify individuals as exposed or unexposed to ice cleat programmes. The number of distributed ice cleat pairs was also used to quantify programme reach. Specifically, we defined reach as the number of distributed pairs per age-eligible citizen using age-and-municipality-specific data from Statistics Sweden’s population register.^13^

#### National surveys

We also used data from existing repeated cross-sectional surveys conducted by Statistics Sweden in 2007, 2010, 2014 and 2018 on behalf of the Swedish Civil Contingencies Agency. In each wave, stratified random samples of the Swedish population aged 18–79 years were invited to answer questionnaires about perceptions and behaviours related to safety and security (*n* respondents=88 676; average response rate across all waves: 52.2%; see online supplemental table S1 for wave-specific data). Register data on municipality of residence, age, sex, place of birth (native, non-native), educational attainment (postsecondary education (>12 years of schooling), secondary education or less (≤12 years of schooling)), was also linked to the respondents via personal identification numbers.^14^ The data were deidentified before being provided to us. Data from the 2018 wave have previously been used to study predictors of ice cleat use in Sweden.^15^ Further details about the survey design are provided in online supplemental appendix.

10.1136/ip-2022-044681.supp1 Supplementary data

### Outcome measure

The outcome variable in this study was self-reported ice cleat use. Each wave of the national surveys contained variations on a question about whether the respondent uses ‘anti-slip protection (eg, ice cleats) on their shoes’ during icy road conditions. The available response categories were on ordinal scales in 2007 and 2010 (from never to always) and on a binary scale (yes or no) in the 2014 and 2018 waves. To homogenise the data, we classified those who reported using ice cleats sometimes, often, or always as ice cleat users in the earlier waves (never or seldom were classified as non-users). The exact phrasing of each question and our coding is presented in online supplemental table S2.

### Exposures

For our primary exposure variable, respondents were classified as exposed if they lived in a municipality with a programme, belonged to an eligible age group and participated in a survey wave after ice cleat distribution. This definition implies an intention-to-treat perspective: All individuals are considered exposed even if they did not participate in the programme, in line with recommendations for estimating real-world effectiveness.^16^ If the municipality had stopped distributing ice cleats, we still classified individuals who fit the above criteria as exposed, because the behaviour change may last longer than the programme.

As a secondary exposure, we defined a continuous exposure variable where we assigned each exposed individual a value corresponding to the number of ice cleat pairs distributed per age-eligible citizen in their municipality (unexposed individuals were assigned a value of zero). This exposure was used to establish a dose–response relationship between programme reach and ice cleat use and to estimate programme efficacy^16^ under ideal conditions, defined here as one pair of ice cleats distributed per age-eligible citizen.

Below, we refer to results for the primary exposure as the association at average reach and the secondary exposure as the association at perfect reach to highlight the difference in interpretation.

### Eligibility and handling of missing data

Respondents living in municipalities that did not respond to our programme survey were excluded due to unknown exposure status (n=25 442). Individuals with missing responses or who responded ‘don’t know’ to the ice cleat questions were treated as non-users (n=3070). Register data on age, sex, place of birth and municipality of residence were complete in all waves. Individuals with missing data on education (n=719) were assigned to the most common category (no postsecondary education).

### Analysis

We first conducted descriptive analyses of the sample characteristics and self-reported ice cleat use by programme exposure status. We used χ^2^ tests and t-tests to assess group differences in categorical and continuous variables, respectively. Associations between the exposure variables and self-reported ice cleat use were then estimated using linear probability models.^17^ The main benefit of this modelling strategy, compared with logistic regression, is that coefficients from linear probability models can directly be interpreted as probability differences. The method also yields unbiased coefficients and consistent SEs in large samples and has been shown to be a more robust choice than logistic regression in models that include many fixed effect terms.^17^ In our fully adjusted specification, we included sex, place of birth (native, non-native), educational attainment (postsecondary education, secondary education or less) and survey wave and municipality fixed effects. In addition to observed individual-level confounding, this model captures unobserved wave-specific effects that are common to all respondents (eg, time trends and the variation in the ice cleat questions) and unobserved effects that are common to individuals living in the same municipality (eg, climate and local injury risks).^18^

All SEs were clustered by municipality, in line with recommendations to cluster at the level at which the intervention is provided.^19^ The analyses were conducted in Stata (V. 17; StataCorp).

#### Subgroup analyses

We conducted subgroup analyses by age group, sex, place of birth and educational attainment to assess potential effect measure modification^20^ by these factors.

#### Sensitivity analyses

We conducted a sensitivity analysis including respondents who reported seldom using ice cleats in the definition of an ice cleat user (see online supplemental table S2). We also considered logistic regression as an alternative to the linear probability models to ensure that the results were not driven by our choice of modelling strategy.

#### Negative control test

We used a negative control group, that is, explored associations among a group that should not be affected by the intervention, to assess the risk of bias.^21^ The negative controls were defined as postintervention respondents between 1 and 15 years younger than the age of eligibility in each programme municipality (65+ years was the most common age of eligibility, so the negative controls mainly consisted of individuals aged 50–64 years). We excluded those above the age of eligibility in programme municipalities to ensure that the negative controls were compared with unexposed respondents from other municipalities (or other ineligible ages within the same municipality). Finally, we repeated the primary statistical analyses as if the negative controls were exposed to programmes, expecting to find null associations.

### Patient and public involvement

No patients or members of the public were involved in the design of the study.

## Results

### Participants

Seventy-three out of the 228 municipalities that responded to our survey responded that they had distributed ice cleats to older adults. We could link programme data from 63 of these programmes to the survey respondents (no one from the remaining ten participated in the surveys after implementation). Of these, 1 had implemented a programme by the 2007 wave, 4 had implemented a programme by 2010, 11 had implemented a programme by 2014 and 63 had implemented a programme by 2018. Regarding eligibility to participate in the programmes, 56 programmes had set the minimum age at 65+ years, 6 had set the minimum age at at 70+ years and 1 had set the minimum age at at 75+years.

After excluding the 25 442 respondents from non-responding municipalities, our final analysis sample contained 63 234 survey participants, of whom 2507 (3.9%) were classified as exposed to ice cleat distribution programmes. The average number of ice cleat pairs distributed per age-eligible citizen was 0.38 (min: 0.01, max: 1.09). In addition, 2192 participants were classified as negative controls (mean age: 57 (min: 50, max: 74)). The participating municipalities and included programme municipalities are listed in online supplemental tables S3 and S4.

### Descriptive data

**Figure 1:**
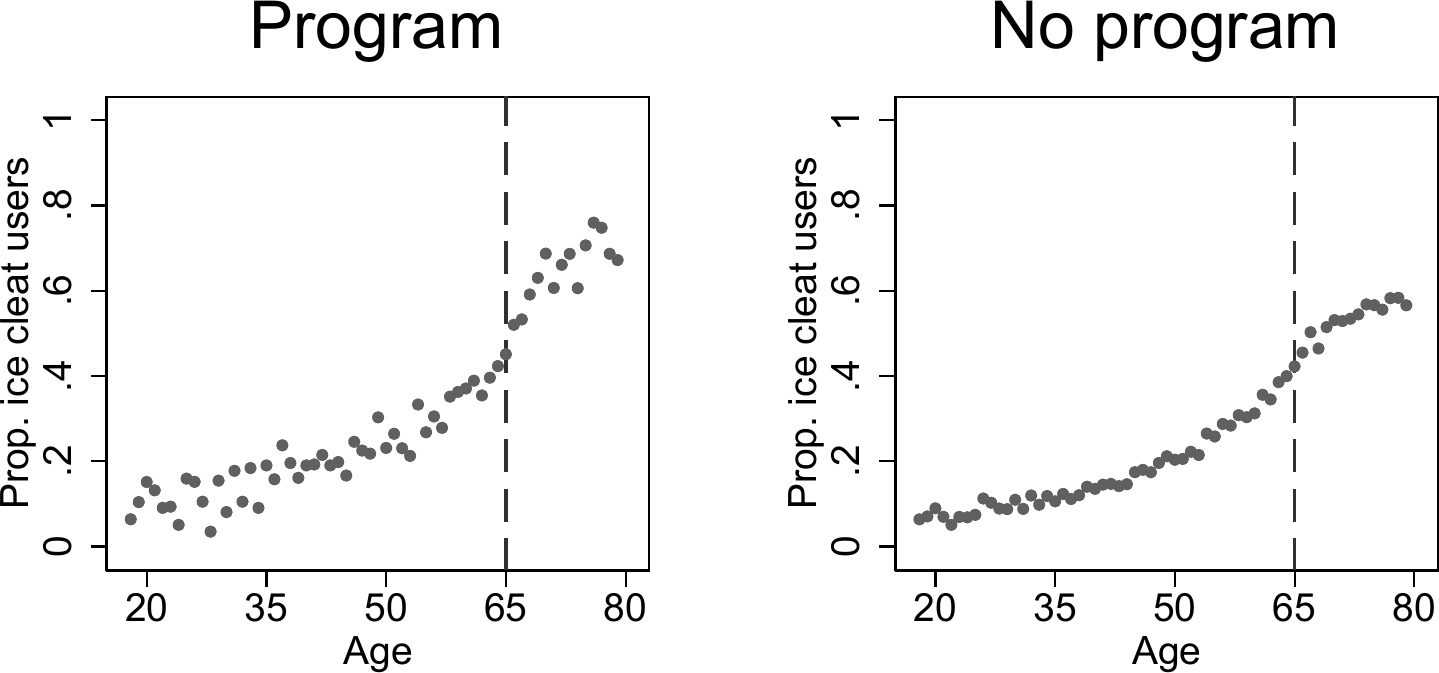
Proportions of self-reported ice cleat users by 1 year age group between 18 and 79 years (n=63 056). Respondents were defined as being from a programme municipality if they lived in a municipality with an ice cleat distribution programme (‘programme’) and responded in a postintervention period; otherwise they were defined as living in a non-programme municipality (‘no programme’). Individuals living in municipalities with minimum eligible age for ice cleat distribution programmes above 65 years of age were excluded from the figures (n=178) to ensure a homogenous age cut-off.

We present the characteristics of all respondents and those aged 65 years and older in table 1, the latter also stratified by programme exposure status. While most characteristics differed significantly (p<0.001) between exposure groups due to the large sample size, we note that the exposure groups were reasonably similar in most characteristics except for survey year (most programmes were implemented late in the study period). Usage rates generally increase with age, but the increase seems to accelerate faster after 65 years in the programme municipalities (figure 1). Overall, older individuals (65+ years) exposed to programmes had a higher ice cleat usage rate (62.9%) than those not exposed to programmes (51.9%) (p<0.001; table 1).

**Table 1:** Characteristics of the sample in total, among older adults, and by exposure to ice cleat distribution programme

Characteristic	All ages	65+ years			
	Entire sample	Entire sample	Unexposed to programme	Exposed to programme	P value*
Sample size—n	63 234	17 906	15 399	2507	
Age—mean (SD)	52.0 (16.7)	71.1 (4.2)	71.1 (4.2)	71.5 (4.1)	<0.001
Women—%	53.1	51.5	51.8	50.3	0.17
No postsecondary education—%	64.9	75.9	76.6	71.4	<0.001
Born in Sweden—%	86.3	88.1	87.7	90.3	<0.001
Survey wave—%					<0.001
2007	18.0	14.0	16.2	0.3	
2010	29.3	24.4	27.7	4.1	
2014	28.3	30.8	34.5	8.0	
2018	24.3	30.8	21.6	87.6	
Ice cleat users, main†—%	29.5	53.5	51.9	62.9	<0.001
Ice cleat users, alternative‡—%	34.0	58.4	57.6	63.7	<0.001
Ice cleat pairs distributed per age-eligible citizen—mean (SD)				0.38 (0.22)	

*P value for group differences in characteristics between exposed and unexposed respondents in the 65+ age group. Based on χ^2^ tests for categorical variables and t-tests for continuous variables.

†Respondents who reported ‘sometimes’, ‘often’ or ‘always’ using ice cleats during icy road conditions were classified as ice cleat users in the waves that used ordinal response scales (2007 and 2010; never or seldom were classified as non-users). Response options to later waves were binary (yes or no).

‡Those who reported seldom using ice cleats were also classified as ice cleat users in this definition.

### Estimates of programme effectiveness at average reach

The age-adjusted ice cleat use was 10.6 (95% CI 5.4 to 15.8) percentage points higher among respondents exposed to the average ice cleat distribution programme than those who were not exposed (table 2, column I). In the fully adjusted model, the probability was 7.5 (95% CI 4.2 to 10.9) percentage higher among exposed respondents than those who were not exposed (table 2, column II).

**Table 2:** Adjusted associations between ice cleat distribution programmes and self-reported ice cleat use, main specifications (columns I–II), sensitivity (columns III–IV) and negative control tests (column V)

	I: Linear probability models, age adjusted	II: Linear probability models, fully adjusted	III: alternative outcome*	IV: Logistic regression models	V: Negative control analysis†
Sample size	63 234	63 234	63 234	63 234	60 727
Primary exposure					
Exposed to programme	0.106 (0.054 to 0.158)	0.075 (0.042 to 0.109)	0.066 (0.034 to 0.099)	0.044 (0.016 to 0.072)	−0.023 (−0.055 to 0.010)
Secondary exposure					
Programme reach‡	0.269 (0.184 to 0.353)	0.173 (0.112 to 0.234)	0.149 (0.090 to 0.208)	0.103 (0.050 to 0.156)	−0.004 (−0.087 to 0.080)

Notes: All models except for column I were fully adjusted for place of birth (native, non-native), educational attainment (postsecondary education, no postsecondary education), sex, age (in 5-year age groups, except 18–24 years), survey and municipality fixed effects. Estimates in cells reflect probability differences in the proportion of ice cleat users between respondents exposed and eligible to a municipal ice cleat distribution programme versus those who were not; 95% CI (clustered by municipality) are presented in parentheses. Coefficients from the logistic regression reflect marginal effects (ie, probability differences) estimated via the margins command in Stata. The primary and secondary exposures were modelled separately.

*Respondents who replied seldom using ice cleats are also considered ice cleat users in this definition.

†Analyses coding respondents between 15 years and 1 year under the age threshold for programmes as the ‘treated’ age group. Sample size is smaller because those who were actually exposed are excluded from the analysis.

‡Distributed ice cleat pairs per age-eligible citizen, coded as zero for unexposed respondents.

### Dose-response and estimates of efficacy at perfect reach

**Figure 2:**
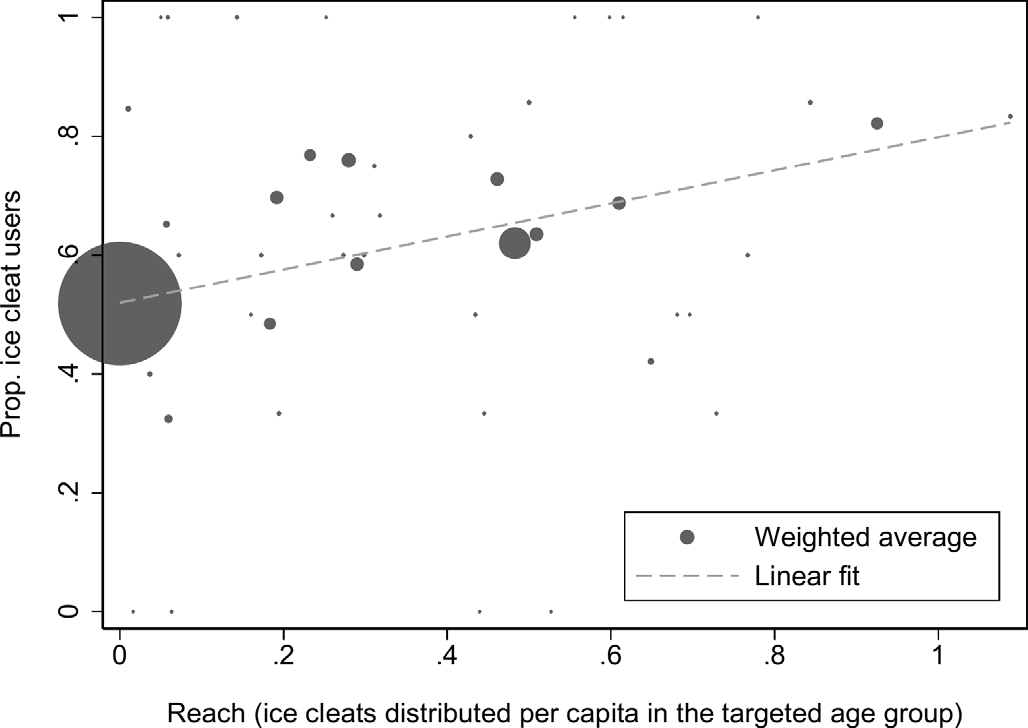
Scatter plot of the average ice cleat usage probability and programme reach for respondents aged 65 years and older. Non-programme municipalities are coded as having zero reach. The size of each circle is proportional to the number of respondents to the national surveys. The average reach in the exposed group was 38%.

The age-adjusted dose-response relationship between ice cleat use and programme reach among older adults (65+ years) showed that greater programme reach was associated with higher ice cleat use (figure 2). Specifically, the estimate implied a 26.9 (95% CI 18.4 to 35.3) percentage point increase at perfect reach (ie, one ice cleat pair distributed per age-eligible citizen) (table 2, column I). The fully adjusted estimate suggested a 17.3 percentage point (95% CI 11.2 to 23.4) increase at perfect reach (table 2, column II).

### Subgroup analyses

The subgroup-specific associations were too imprecise to establish significant effect measure modifiers, as all 95% CIs overlap (table 3). However, the point estimates suggested the following patterns: (1) the associations grew stronger with older age, (2) the association was stronger for men than women, (3) the association was similar in the two education groups and (4) the association was stronger for non-natives than natives.

**Table 3:** Subgroup analyses of the proportion of ice cleat users among unexposed and exposed respondents and the adjusted association between ice cleat distribution programmes and self-reported ice cleat use based on linear probability models

Subgroup	Proportion of ice cleat users among the unexposed (65+ years)	Proportion of ice cleat users among the exposed (65+ years)	Adjusted probability difference (exposed vs unexposed)	Sample size
Age group				
65–69 years	0.471	0.546	0.034 (−0.024 to 0.093)	7255
70–74 years	0.541	0.650	0.053 (−0.006 to 0.112)	6197
75–79 years	0.570	0.716	0.091 (0.025 to 0.157)	4454
Sex				
Men	0.402	0.526	0.101 (0.057 to 0.145)	29 634
Women	0.628	0.732	0.053 (0.018 to 0.089)	33 600
Educational attainment				
Postsecondary education	0.553	0.644	0.065 (0.007 to 0.124)	22 190
Secondary education or lower	0.509	0.624	0.076 (0.042 to 0.109)	41 044
Place of birth				
Sweden	0.534	0.633	0.069 (0.034 to 0.104)	54 582
Other	0.417	0.597	0.109 (0.035 to 0.184)	8652

Notes: Analyses were adjusted for place of birth (native, non-native), educational attainment (postsecondary education, no postsecondary education), sex, age (in 5-year age groups, except 18–24 years), survey and municipality fixed effects. Cluster-robust 95% CI (clustered by municipality) are presented in parentheses. The subgroups were modeled separately.

### Sensitivity analyses

Sensitivity analyses using the alternative definition of ice cleat users and logistic regression showed slightly smaller associations, but the conclusions remain the same (table 2, columns III and IV).

### Negative control test

The negative control analyses showed no associations between ice cleat distribution and ice cleat use among younger, ineligible individuals living in programme municipalities (table 2, column V).

## Discussion

This is the first study to assess the association between ice cleat distribution programmes and ice cleat use. Our results are consistent with the hypothesis that ice cleat distribution increases use: We found an association between programme exposure and usage rates, which was stronger in municipalities that distributed more ice cleats per age-eligible citizen, implying a dose–response relationship. We also found suggestive evidence association may be stronger in groups with lower baseline use (especially men and non-native Swedes; table 3), indicating that municipal ice cleat distribution may serve as a tool to decrease inequality in personal safety. However, the subgroup estimates were too imprecise to draw strong conclusions about effect measure modification.

As noted in the introduction, it is relatively well established that using ice cleats can reduce the risk of ice-related injuries at the individual level.^5–7 10^ Only a few studies have investigated ice cleat distribution programmes.^8 11 12^ Of these, a process evaluation study found that Swedish ice cleat programmes reached about 40% of their target population regarding the number of distributed ice cleats, with limited evidence of severe implementation issues.^8^ An impact evaluation also found that pedestrian fall injuries related to snow and ice decreased among older adults in Gothenburg following the introduction of their ice cleat programme.^11^ However, none have previously been able to establish an association with increased ice cleat use.

Together with this study, our interpretation is that the current research supports the idea that ice cleat distribution may be an effective injury prevention programme in places affected by icy road conditions, especially when considering the cost-effectiveness of these devices. A model-based economic evaluation that combined effect estimates from a randomised trial^7^ and cost data from Swedish municipalities also showed that a 0.15 percentage point increase in ice cleat use would be sufficient for the programmes to be cost–beneficial from a Swedish societal perspective if one pair of ice cleats is purchased per age-eligible citizen^12^; an estimate that is far below the association we observed (7.5 percentage points at average reach; 17.3 at perfect reach).

### Strengths and limitations

A weakness of our study is that we could only make ecological connections between individuals and ice cleat distribution programmes.^22^ Consequently, we cannot confirm that the observed associations are driven by individuals who took part in the ice cleat distribution.

Additionally, our observational approach is prone to confounding bias. To assess the risk of bias, we used younger, ineligible individuals within the same municipality as negative controls.^21^ We found no association between programme exposure and ice cleat use among the negative controls, implying that the results are not driven by age-independent confounders that set programme municipalities apart from the other municipalities in the data. However, this test cannot identify if age-eligible individuals in programme municipalities systematically differ from unexposed individuals of the same age in control municipalities.

Our exposures may also be susceptible to misclassification^23^ if the municipalities that answered our survey reported erroneous programme data. We believe that our secondary exposure (distributed ice cleats per capita) is more susceptible to this issue than the primary (yes or no) exposure, as documentation of the number of distributed ice cleats may be inadequate. Further, our outcome variable is self-reported and may be prone to bias if exposed individuals feel the need to falsely report using ice cleats because of the programmes.^24^ However, we note that there is no direct connection between the questions asked in the national surveys and the distribution programmes, so this concern should be limited.

We used repeated cross-sectional data from national samples, enhancing generalisability to the Swedish population. However, selective participation is always a concern despite using a random sampling design.^25^ Moreover, the surveys only sampled individuals below 80 years, so we could not assess associations for individuals over that age. It is also unclear if our results are transferable to other settings. At the very least, we hope that they can provide suggestive data for decisions about interventions in other countries with icy winter conditions with similar populations and local government structures as Sweden.

## Conclusions

Distributing ice cleats to older adults in settings affected by icy road conditions may help increase ice cleat use. Confirmatory research with randomised distribution would help further establish a causal relationship between ice cleat distribution and changes in ice cleat use, and empirical research on reductions in ice-related fall injury rates is required to confirm the cost-effectiveness of the existing ice cleat programmes in Sweden.

## Data Availability

Data are available on reasonable request. Data may be obtained from a third party and are not publicly available. This study used data from two sources. Our programme survey data are non-sensitive and will be shared with anyone upon reasonable request. The national survey data are protected by a confidentiality agreement with the Swedish Civil Contingencies Agency and therefore cannot be shared publicly. Researchers interested in obtaining these data can contact the Swedish Civil Contingencies Agency.
