# Supplementary material for: Ice cleat distribution programmes and ice cleat use among older adults: repeated cross-sectional evidence from 63 municipal interventions in Sweden
Source: Inj Prev. 2022 Aug 3;28(6):539–44. doi: 10.1136/ip-2022-044681 (PMC9726961; doi:10.1136/ip-2022-044681)
Supplement: Supplementary data [file ip-2022-044681supp001.pdf]

Online Appendix for ”Ice cleat distribution programs and ice cleat use among older adults: repeated cross-sectional evidence from 63 municipal interventions in Sweden”

Online Supplementary Appendix

This Appendix contains supplementary information about data collection methods and supplementary tables.

Contents

Data collection, additional details 1

Electronic municipal survey to collect data on ice cleat distribution programs 1

Statistics Sweden surveys to collect data on ice cleat use 2

Measurement of ice cleat use per survey wave 3

Supplementary tables 5

Data collection, additional details

Electronic municipal survey to collect data on ice cleat distribution programs

We designed an electronic survey sent to all municipalities in Sweden (*n* = 290) on June 10th, 2019 (with up to four reminders sent on July 1st, 2019; August 16th, 2019; September 10th, 2019; and October 16th, 2019). The survey collected information about the ice cleat programs, e.g. if the municipalities ever had or have an ongoing ice cleat distribution program, the time span of the programs (when they were introduced and/or ended), the amount of distributed ice cleats, the costs of the programs, the targeted age group, etc. The municipal respondents were also given the opportunity to reply with supplementary information via e-mail. In total, 228

Online Appendix for ”Ice cleat distribution programs and ice cleat use among older adults: repeated cross-sectional evidence from 63 municipal interventions in Sweden”

municipalities participated in the survey. During the data retrieval process, the region of Jönköping informed us that they acted as the main distributor of ice cleats in their region (the Swedish municipalities are divided into 21 regions). This affected a total of 13 municipalities. Nine of them had already responded to the survey, which made us add additional four municipalities as exposed to a program. Our study focuses on municipalities that have introduced distribution programs targeting older adults. Therefore, five municipalities were excluded as they distributed ice cleats to all ages, making them ineligible for analysis. In summary, a total of 227 municipalities were included in the program data that we matched to respondents from Statistics Sweden’s surveys (see next section). Respondents from 223 of these municipalities were available in the survey data. The number of survey respondents per municipality is presented in Table S3. A corresponding list, containing only the 63 matched municipalities with programs, is available in Table S4.

Statistics Sweden surveys to collect data on ice cleat use

In recent decades, the Swedish Civil Contingencies Agency has repeatedly commissioned Statistics Sweden to investigate how the Swedish population perceives how safe their everyday lives are. To do this, Statistics Sweden designed nationwide surveys using a stratified random sampling design, targeting adults living in Sweden aged 18-79 (about 7 million people). The surveys were designed to investigate individuals’ self-reported perceptions of everyday threats and risks and their perceptions of safety and security. Also, Swedish municipalities were allowed to purchase a municipality-specific survey in addition to the national data collected by Statistics Sweden (600 survey samples per municipality, per year). Four nationwide survey waves were conducted in 2007, 2010, 2014, and 2018, with new random samples in each wave (i.e., the data does not contain repeated observations on the same individuals). Random samples stratified by age group, sex, place of birth (in the

Online Appendix for ”Ice cleat distribution programs and ice cleat use among older adults: repeated cross-sectional evidence from 63 municipal interventions in Sweden”

2007, 2010 waves) and age group and sex (in the 2014, 2018 waves) were drawn from a national sampling frame. Respondents within a stratum had the same probability of being included in the sample. Measures were applied to not double-count survey participation (the same participants who responded to the national survey could not participate in the municipal survey). Statistics Sweden also linked data on educational attainment (from the Swedish Education Register) to the respondents using personal identification numbers. In summary, 169,721 respondents were asked to participate in the surveys (see Table S1 for details), and a total of 88,676 respondents participated (i.e., a 52.2% response rate).

Measurement of ice cleat use per survey wave

In every survey conducted by Statistics Sweden, there were variations on questions relating to the use of personal safety equipment, and each survey included subquestions related to ice cleats (see Table S2 for details). However, there were differences in outcome responses in the four waves that needed to be handled to make them more homogeneous for analysis (the last two waves only included a yes or no question). In the first two waves (2007, 2010), the respondents were asked: “How often do you do the following for your own safety?”, with subquestions “Use anti-slip protection when the roads are icy (e.g., ice cleats)” (in 2007) and “Use anti-slip protection on your shoes (e.g., ice cleats) when it is slippery or icy” (in 2010).

In the first survey (year 2007), the respondents were given six response options, using an ordinal scale with alternatives ranging from; (1) *never*, (2) *seldom*, (3) *sometimes*, (4) *often*, (5) *always*, and (6) *don’t know* (total participants *n* = 11,186*)*. For our primary analysis, we dichotomized the self-reported outcomes defining alternatives 3-5 as ice cleat users (*n* = 2,595), while using 1, 2 and 6, the *never* (*n* = 7,112), *seldom* (*n* = 971) and *don’t know*-users

Online Appendix for ”Ice cleat distribution programs and ice cleat use among older adults: repeated cross-sectional evidence from 63 municipal interventions in Sweden”

(*n* = 508) as non-ice cleat users. To assess the implications of this interpretation, we also conducted a sensitivity analysis by recoding the *seldom*-users as ice cleat users.

The second survey (year 2010) reduced the number of self-rated alternatives to using ice cleats from six to five; (1) *never or very rarely*, (2) *sometimes*, (3) *often*, (4) *always or almost always*, (5) *don't know* (total participants *n* = 18,546). Once again, we recoded and dichotomized the reported outcomes defining options 3 and 4 as ice cleat users (*n* = 3,082), using the remaining alternatives *never or very rarely*-users (*n* = 12,112), and *don't know*-users (*n* = 1,173) as non-users. We also coded the *sometimes*-users (*n* = 1,872) as ice cleat users in a sensitivity analysis.

The other two survey samples, the years 2014 (*n* = 17,916) & 2018 (*n* = 15,362), Statistics Sweden asked this question differently; "*Do you use any of the following safety equipment?*" with five sub-questions and we addressed the ice cleat-question specifically; "*Do you use anti-slip protection on your shoes (e.g., ice cleats) when it is slippery or icy outside?*". The answer options were binary coded: (1) yes, (2) no, and (3) don't know. The respondents who stated that they use ice cleats (1) are used for the primary analyses (year 2014 *n* = 6,425 & year 2018 *n* = 6,536), and non-users (2) and don't know users (3) were coded as non-users (year 2014 *n* = 11,491 & year 2018 *n* = 8,826).

Online Appendix for ”Ice cleat distribution programs and ice cleat use among older adults: repeated cross-sectional evidence from 63 municipal interventions in Sweden”

Supplementary tables

Table S1. Sample size and response rate of surveys conducted by Statistics Sweden on behalf of the Swedish Civil Contingencies Agency.

		Survey year			
		**2007**	**2010**	**2014**	**2018**
Sample size					
	National sample	12 000	10 000	10 000	10 000
	Municipality samples	21 600	37 800	34 800	33 521
	Total	33 600	47 800	44 800	43 521
Response rate (%)					
		20 881 (62.1%)	26 161 (54.7%)	23 168 (51.7%)	18 466 (42.4%)
	Municipality[a]	55-70%	44-62%	44.5-62.2%	38-46%

a Shows the interval for the response rate for the municipality-specific survey. The number of municipalities that purchased survey participation varies each survey year.

Online Appendix for ”Ice cleat distribution programs and ice cleat use among older adults: repeated cross-sectional evidence from 63 municipal interventions in Sweden”

Table S2. Questions and response categories relating to ice cleat use in each wave (in Swedish and with our translation to English), and coding rules for the main outcome measure and sensitivity outcome measure.

	Original question (in Swedish)		English translation		Coding	
Wave	Question	Response categories	Question	Response categories	Main analysis	Sensitivity analysis
2007	”Hur ofta gör du nedanstående saker för din egen säkerhets skull?” *(Main question)* + ” Använder halkskydd vid halt väglag (t.ex. broddar)”	Aldrig (1); Sällan (2); Ibland (3); Ofta (4); Alltid (5); Ej aktuellt (6).	”How often do you do the following for your own safety?” (Main question) + “Use anti-slip protection when the roads are icy (e.g., ice cleats)”	Never (1); Seldom (2); Sometimes (3); Often (4); Always (5); Not relevant (6).	ICE CLEAT USER = YES IF 4 OR 5, ELSE NO (MISSING AS NO).	ICE CLEAT USER = YES IF 3 OR 4 OR 5, ELSE NO (MISSING AS NO).
2010	”Hur ofta gör du följande för din egen säkerhets skull?” *(Main question)* + ”Använder halkskydd på skorna (t.ex. broddar) när det är halt eller isigt” *(Subquestion)*	Aldrig eller mycket sällan (1); Ibland (2); Ofta (3); Alltid eller nästan alltid (4); Vet inte/ej aktuellt (5).	”How often do you do the following for your own safety?” (Main question) + “Use anti-slip protection on your shoes (e.g., ice cleats) when it is slippery or icy” (Subquestion)	Never or very rarely (1); Sometimes (2); Often (3), Always or almost always (4); Don’t know/not relevant (5)	ICE CLEAT USER = YES IF 3 OR 4, ELSE NO (MISSING AS NO).	ICE CLEAT USER = YES IF 2 OR 3 OR 4, ELSE NO (MISSING AS NO).
2014	”Använder du någon av följande säkerhetsutrustning?” *(Main question)* + ”Använder du halkskydd på skorna (t.ex. broddar) när det är halt eller isigt ute?” *(Subquestion)*	Ja (1); Nej (2); Vet ej (3).	”Do you use any of the following safety equipment?” (Main question) + “Do you use anti-slip protection on your shoes (e.g., ice cleats) when it is slippery or icy outside?”	Yes (1); No (2); Don’t know (3).	ICE CLEAT USER = YES IF 1, ELSE NO (MISSING AS NO).	SAME AS MAIN.
2018	”Använder du någon av följande säkerhetsutrustning?” *(Main question)* + ”Använder du halkskydd på skorna (t.ex. broddar) när det är halt eller isigt ute?” *(Subquestion)*	Ja (1); Nej (2); Vet ej (3).	”Do you use any of the following safety equipment?” (Main question) + “Do you use anti-slip protection on your shoes (e.g., ice cleats) when it is slippery or icy outside?”	Yes (1); No (2); Don’t know (3).	ICE CLEAT USER = YES IF 1, ELSE NO (MISSING AS NO).	SAME AS MAIN.

Online Appendix for ”Ice cleat distribution programs and ice cleat use among older adults: repeated cross-sectional evidence from 63 municipal interventions in Sweden”

Table S3. Municipalities that participated in the electronic survey that could be matched to the national surveys on ice cleat use (n=223), and the number of survey participants per municipality, age 18-79 (n=63,234)

Municipality	*Survey responses*	*Municipality*	*Survey responses*	*Municipality*	*Survey responses*
Alvesta	386	Karlstad	867	Stenungsund	394
Aneby	19	Kil	344	Storfors	8
Arjeplog	4	Klippan	34	Storuman	17
Arvidsjaur	14	Kristinehamn	343	Strängnäs	79
Arvika	711	Krokom	32	Strömstad	43
Askersund	319	Kumla	15	Strömsund	23
Avesta	25	Kungsör	21	Sundbyberg	79
Bengtsfors	324	Kungälv	762	Sunne	31
Berg	16	Kävlinge	404	Surahammar	29
Bjurholm	308	Köping	56	Svedala	38
Bjuv	39	Laholm	393	Svenljunga	18
Bollebygd	21	Laxå	651	Säffle	605
Bollnäs	47	Lekeberg	8	Säter	12
Borgholm	370	Leksand	391	Sävsjö	26
Borlänge	56	Lessebo	173	Södertälje	67
Borås	643	Lidingö	385	Tanum	32
Botkyrka	483	Lidköping	54	Tidaholm	9
Bräcke	20	Lilla Edet	338	Tierp	342
Burlöv	47	Linköping	1,218	Timrå	91
Båstad	38	Ljungby	66	Tjörn	375
Dals-Ed	7	Ljusdal	21	Tomelilla	39
Danderyd	378	Ljusnarsberg	5	Torsby	26
Dorotea	1	Lomma	422	Torsås	383
Eda	574	Ludvika	28	Tranås	697
Ekerö	352	Luleå	1,181	Trelleborg	395

Online Appendix for ”Ice cleat distribution programs and ice cleat use among older adults: repeated cross-sectional evidence from 63 municipal interventions in Sweden”

Eksjö	406	Lund	251	Trollhättan	461
Emmaboda	19	Lycksele	337	Trosa	16
Enköping	44	Lysekil	209	Tyresö	69
Eskilstuna	550	Malmö	1,388	Töreboda	15
Eslöv	63	Malå	8	Uddevalla	811
Fagersta	564	Mariestad	35	Ulricehamn	56
Falkenberg	1,069	Mark	450	Umeå	1,308
Falköping	384	Mellerud	22	Upplands-Bro	38
Filipstad	21	Mjölby	36	Uppsala	466
Finspång	719	Mora	386	Vadstena	10
Flen	434	Mullsjö	740	Vaggeryd	659
Forshaga	332	Munkfors	278	Valdemarsvik	13
Färgelanda	14	Mölndal	803	Vansbro	6
Gislaved	27	Mönsterås	29	Vara	22
Gnosjö	12	Mörbylånga	38	Varberg	1,572
Gotland	486	Nordanstig	75	Vaxholm	24
Grums	312	Nordmaling	14	Vetlanda	946
Grästorp	9	Norrköping	1,122	Vimmerby	29
Gullspång	8	Norsjö	4	Vingåker	455
Gällivare	314	Nybro	420	Vänersborg	92
Gävle	340	Nykvarn	9	Vännäs	394
Göteborg	4,374	Nässjö	913	Värmdö	18
Habo	1,515	Ockelbo	17	Värnamo	664
Hagfors	23	Olofström	40	Västervik	489
Hallsberg	11	Orsa	5	Västerås	330
Halmstad	1,25	Orust	19	Växjö	575
Hammarö	375	Osby	31	Vårgårda	13
Haninge	123	Oskarshamn	55	Ydre	774

Online Appendix for ”Ice cleat distribution programs and ice cleat use among older adults: repeated cross-sectional evidence from 63 municipal interventions in Sweden”

Haparanda	29	Ovanåker	13	Ystad	64
Hedemora	16	Oxelösund	25	Älmhult	413
Helsingborg	686	Pajala	3	Älvkarleby	38
Herrljunga	17	Piteå	774	Älvsbyn	18
Hjo	18	Ronneby	165	Ängelholm	78
Hofors	35	Sala	1,087	Åmål	356
Hultsfred	33	Salem	28	Ånge	22
Hylte	18	Sandviken	115	Åre	27
Hällefors	6	Sigtuna	601	Årjäng	18
Härjedalen	33	Simrishamn	51	Åstorp	31
Härryda	455	Sjöbo	46	Åtvidaberg	11
Hässleholm	100	Skara	23	Öckerö	719
Håbo	17	Skellefteå	1,045	Örebro	640
Högsby	12	Skinnskatteberg	10	Örkelljunga	22
Hörby	27	Skurup	43	Örnsköldsvik	1,559
Jokkmokk	17	Skövde	95	Östersund	560
Järfälla	100	Smedjebacken	15	Österåker	77
Jönköping	1,739	Sollefteå	41	Östhammar	2
Kalmar	548	Sollentuna	428	Östra Göinge	30
Karlsborg	4	Solna	164	Övertorneå	15
Karlskoga	21	Sorsele	4		
Karlskrona	673	Sotenäs	25		

Online Appendix for ”Ice cleat distribution programs and ice cleat use among older adults: repeated cross-sectional evidence from 63 municipal interventions in Sweden”

Table S4. The municipalities that implemented ice cleat distribution programs for older adults (n=63) and the number of survey participants per municipality, ages 65-79 and exposed to ice cleat distribution (n=2.507).

Municipality	***Survey responses***	***Municipality***	***Survey responses***	***Municipality***	***Survey responses***
Aneby	2	Jönköping	133	Sundbyberg	5
Askersund	2	Kalmar	10	Svenljunga	1
Bengtsfors	1	Karlskrona	130	Säffle	132
Borgholm	4	Krokom	1	Säter	4
Borås	7	Kungsör	2	Sävsjö	6
Dorotea	1	Laholm	6	Tranås	118
Eksjö	3	Laxå	154	Trollhättan	16
Emmaboda	1	Lidingö	5	Töreboda	3
Fagersta	101	Lidköping	13	Uddevalla	95
Gislaved	2	Ljusdal	4	Uppsala	130
Gnosjö	1	Lund	37	Vaggeryd	122
Grästorp	1	Mark	137	Valdemarsvik	3
Gällivare	3	Mellerud	1	Vetlanda	153
Göteborg	128	Mullsjö	1	Värnamo	131
Habo	130	Mönsterås	3	Västervik	6
Halmstad	19	Norrköping	136	Älmhult	1
Haninge	23	Nässjö	106	Åmål	136
Haparanda	4	Oskarshamn	5	Öckerö	2
Härryda	97	Skövde	5	Örkelljunga	1
Hörby	3	Smedjebacken	1	Österåker	7
Järfälla	5	Strängnäs	5	Övertorneå	2
